# What the hyena's laugh tells: Sex, age, dominance and individual signature in the giggling call of *Crocuta crocuta*

**DOI:** 10.1186/1472-6785-10-9

**Published:** 2010-03-30

**Authors:** Nicolas Mathevon, Aaron Koralek, Mary Weldele, Stephen E Glickman, Frédéric E Theunissen

**Affiliations:** 1 Université de Saint-Etienne, Equipe 'Neuro-Ethologie Sensorielle', CNPS, CNRS UMR 8195, Saint-Etienne, France; 2 Centre National de la Recherche Scientifique, CNPS, UMR 8195, France; 3 University of California at Berkeley, Helen Wills Neuroscience Institute, Berkeley, USA; 4 University of California at Berkeley, Department of Psychology, Berkeley, USA; 5 University of California at Berkeley, Department of Integrative Biology, Berkeley, USA

## Abstract

**Background:**

Among mammals living in social groups, individuals form communication networks where they signal their identity and social status, facilitating social interaction. In spite of its importance for understanding of mammalian societies, the coding of individual-related information in the vocal signals of non-primate mammals has been relatively neglected. The present study focuses on the spotted hyena *Crocuta crocuta*, a social carnivore known for its complex female-dominated society. We investigate if and how the well-known hyena's laugh, also known as the giggle call, encodes information about the emitter.

**Results:**

By analyzing acoustic structure in both temporal and frequency domains, we show that the hyena's laugh can encode information about age, individual identity and dominant/subordinate status, providing cues to receivers that could enable assessment of the social position of an emitting individual.

**Conclusions:**

The range of messages encoded in the hyena's laugh is likely to play a role during social interactions. This call, together with other vocalizations and other sensory channels, should ensure an array of communication signals that support the complex social system of the spotted hyena. Experimental studies are now needed to decipher precisely the communication network of this species.

## Background

The origin and maintenance of social group structure is a topic of central concern in vertebrate biology [1-4]. Whereas one approach is to understand the processes that can account for sociality over an evolutionary scale [5-8], a proximal point of view aims to decipher the mechanisms by which the social structure of a group is maintained -or not- over an individual lifetime scale [9-13]. Since Darwin's book on the expression of emotion [14] and followed by numerous studies in the field, it is well known that information on social status can help individuals adjust their behaviour, for example by avoiding useless fights and polishing social interactions [15-17]. Chemical, visual and acoustic signals have been shown to encode information about sex, kinship, individual identity, morphological cues, as well as motivational and physiological states of the sender [18-28]. As some of these cues can potentially be correlated to fighting ability and dominance rank, the information helps congeners evaluate the emitter's social position within the group [18,29-38]. We extended these studies by examining the information that is present in one of the acoustic communication signals of a unique social carnivore, the spotted hyena *Crocuta crocuta*. Spotted hyenas are nocturnal social carnivores, typically living in multi-male, multi-female, "clans" of 10 - 90 individuals. Spotted hyenas are efficient hunters. A lone hyena is capable of capturing prey as large as a wildebeest. Hyenas will also hunt collaboratively, for example to catch zebras [39]. But both sole and collaborative hunting can generate intense competition as clan mates will converge on the carcass. Spotted hyenas have a matrilineal social system similar to that of many old world primates [40]. Within spotted hyena clans, there are separate male and female dominance hierarchies, but all females, and their sub-adult offspring, totally dominate all adult immigrant males. Such female dominance persists in the captive colony at the University of California, Berkeley, where we conducted this study. This colony was established in order to permit the study of the endocrine substrates of dominance and aggression, as well as the basis of "masculinization" of the external genitalia of female spotted hyena, which occurs *in utero *[41].

In nature, the dominance hierarchies described above determine priority of access to food. The hierarchical position of an individual is fully acquired around 18 months of age and is not correlated with size or fighting ability: the social status of females is determined by their mothers' social rank [42-44], whereas males' one depends on their sequence of arrival within the social group [45,46]. During their entire life, hyenas form coalitions against competitors to defend their social ranks [47]. Within their clan, individuals have to cope with a complicated network of social congeners, and this promotes the development of effective abilities to discriminate and rank congeners. Although, it is known that hyenas communicate through visual, chemical and acoustical modalities, there is still much to learn about the nature of exchanged information and the way it is encoded into communication signals [48].

One of the most striking hyena communication channels is acoustical [39]. These animals are well known for their vocalizations that dominate the nightly soundscape in the African savannah. The vocal repertoire of the spotted hyena is large, with more than ten different vocalizations, many of them being graded into each other which makes them difficult to be classified [39,49]. As hyenas are primarily nocturnal [50], vocal signalling is a privileged channel, used for both long- and short-range communications. For instance, "whoops," with long inter-whoop intervals, are primarily used to signal separated individuals, supporting within- and between-clans acoustic interaction [51,52]; conversely, the "grunt" [39] ("soft growl" in [49]) is uttered during close meeting of clan mates and remains barely audible after a few meters of propagation. Previous studies have focused on the long-distance "whoop" call, showing that it supports information related to sex and individual identity and thus may allow discrimination between clan members and alien individuals [48,51,53]. Although they might play an important role in mediating the relationships within the clan, calls, other than the whoop, of adult hyenas have been neglected. Although inferences have been drawn from the contexts in which the various vocalizations are emitted, the information that such vocalizations contain has not yet been investigated [48].

**Figure 1:**
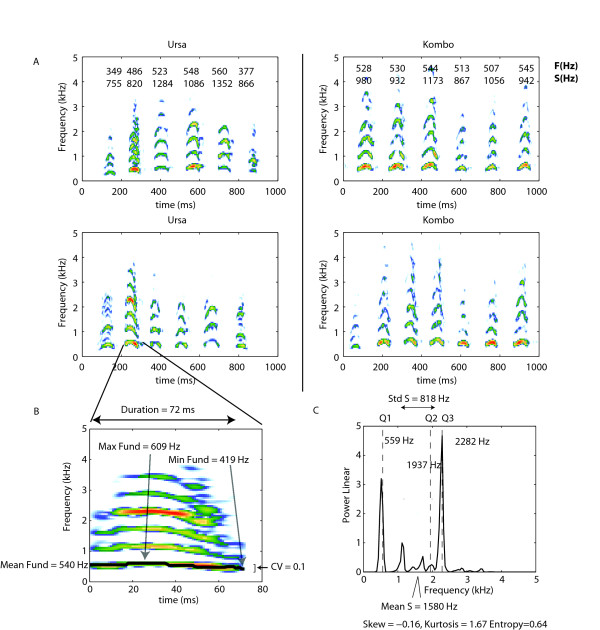
**Acoustic structure of the hyena's giggle (laughing) call**. **A**. Two giggle bouts emitted by two different individuals (Ursa and Kombo). The mean fundamental F(Hz) and the spectrum mean S(HZ) for each giggle note is shown on top of its spectrographic representation for the giggle bouts in the first row. Ursa is a subordinate animal while Kombo is a dominant animal. Ursa is a 10 yr old female and Kombo is a 9 yr old female. They are both control animals (no hormonal treatment). The sounds of the giggle bouts for Ursa are available as Additional Files 1 and 2. The sounds of the giggle bouts for Kombo are available as Additional Files 3 and 4. **B**. Spectrogram of the second giggle note from the bout for Ursa shown in the lower panel. The fundamental frequency is underlined in black. From this time varying fundamental frequency, we extracted the mean, the minimum and maximum values as well as the coefficient of variation (CV). The values for this note are shown on the plot. **C**. Frequency spectrum of the giggle note shown in B. From the frequency spectrum, we obtained the mean frequency, standard deviation, skew, kurtosis, entropy and the three frequency values that delineate the quartiles in energy. The values for this note are shown on the plot (see main text for additional details about fundamental calculation and measurements of acoustic parameters)

Among the vocalizations used during interactions between adult clan-mates, we chose to focus on the "giggle call", often referred as the *hyena's laugh *(Figures 1 (see additional files 1, 2, 3 &4 for sound) 2 (see additional files 5, 6, 7 &8 for sound); [39]). Giggles are high pitched sounds emitted in bouts, mainly when hyenas are feeding together on a prey [39,49]. This call has been described by field observers as a submissive vocalization uttered by an individual in front of a dominant [39,49]. Although giggles are emitted during close-range interactions between two or more individuals, they are loud and can be easily eavesdropped by other clan-mates. In nature, giggles are commonly emitted during competitions between dominant and subordinate animals such as those that would occur for food at a carcass [39,49], but identification of individual giggles by human observers is difficult because of the simultaneous emission of such giggles by multiple individuals. In the Berkeley colony, it was possible to record giggles emitted by individual hyenas when presented with food. In the present report, we describe the use of acoustic structure analysis, in order to determine whether the giggle of spotted hyenas encodes information about sex, age, dominance and individual identity, and if it could allow congeners to assess an emitter's social status based on its laughing cues.

**Figure 2:**
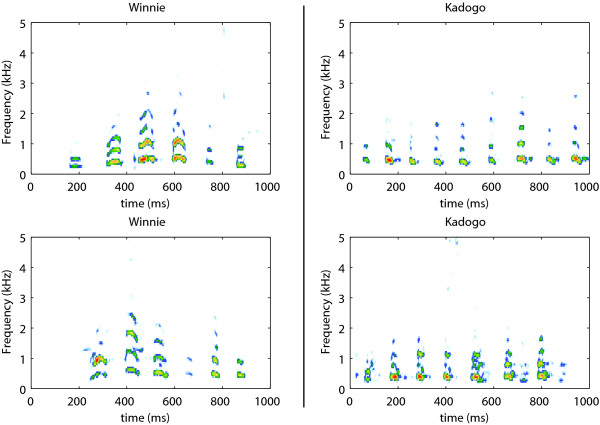
**A second example of the hyena's giggle call for two experimental animals**. A majority of animals used in this study were treated with hormones or gonadectomized as participants in other research projects. In this figure, we show two giggle bouts emitted by two different individuals, Winnie and Kadogo. Winnie is a 14 yr old male treated with anti-androgens in utero. Kadogo is a 6 year old female treated with anti-oestrogens in utero. Hormonal treatment did not have an effect on the acoustical structure of the giggle notes or giggle bouts as assessed by our measures. Winnie is a subordinate animal while Kadogo is a dominant animal. The sounds of the giggle bouts for Winnie are available as Additional Files 5 and 6. The sounds of the giggle bouts for Kadogo are available as Additional Files 7 and 8.

## Methods

### Subjects

The animals that participated in the study are members of a captive breeding colony of spotted hyenas, maintained at the Field Station for the Study of Behavior, Ecology, and Reproduction (FSSBER) at the University of California, Berkeley. Currently, this colony houses 26 hyenas: 14 adult females, 10 adult males, and 2 sub-adult males (less than two years of age at the time of testing). Animals are sexed and their life history is known. The reproductive founders of the Berkeley colony were collected in 1985 in the Narok district of Kenya. The present study was conducted in September-October 2008, and all the animals currently at the field station were born in captivity. The FSSBER is fully accredited by the American Association for the Accreditation of Laboratory Animal Care and the animal care also met all institutional guidelines. The behavioural procedures were approved by the Institutional Animal Care and Use Committee (IACUC) of the University of California, Berkeley.

In the field station, individuals are housed in groups of two (n = 7 dyads) or three (n = 1 one triad) in outdoor or semi-outdoor enclosures (enclosure mean area = 330 m^2^, min-max = 147-929 m^2^). Table 1 provides detailed for information about individuals whose giggles were analyzed in the present report. The dominance status within each dyad was assessed on a daily basis by caretakers familiar with the animals. There was perfect agreement between two caretakers who independently assigned dominance ranks, based upon observation of submissive (e.g., retreat, tail between legs) and aggressive acts (e.g., push, stand over, chase). For each dyad, one animal was qualified as 'dominant', the other as 'subordinate' (Table 1).

**Table 1:** List and characteristics of recorded hyenas.

Animal name and number	Sex	Age (years)	Treatment	Experimental group	Dominant (D)/Subordinate (S)	Number of analysed giggle notes
#30 Rocco (lives with Domino)	male	20	vasectomized	Control	S	20

#57 Gremlin (lives with Kadogo)	male	14	Flut	Anti-androgen	S	98

#59 Winnie (lives with Kombo)	male	14	Flut&Fin	Anti-androgen	S	40

#64 Dusty (lives with Denali)	male	11	gonadectomized	gonadectomized	D	35

#65 Denali (lives with Dusty)	male	11	Control	Control	S	27

#82 Buster (lives with BJ)	male	7	Letrozole	Anti-oestrogen	S	52

#55 Nakuru (lives with Nairobi)	female	14	Flut&Fin	Anti-androgen	S	26

#56 Nairobi (lives with Nakuru)	female	14	Flut&Fin	Anti-androgen	D	31

#61 Domino (lives with Rocco)	female	13	Flut&Fin	Anti-androgen	D	63

#63 BJ (lives with Buster)	female	12	gonadectomized	gonadectomized	D	33

#68 Ursa (lives with Cass)	female	10	Control	Control	S	53

#71 Cass (lives with Ursa)	female	10	Control	Control	D	55

#77 Kombo (lives with Winnie)	female	9	Control	Control	D	42

#83 Kadogo (lives with Gremlin)	female	6	Letrozole	Anti-oestrogen	D	33

#90 Tembo (lives with Haji and Jambo)	male	2	Fin&Csdx	Anti-androgen	S	26

#91 Haji (lives with Tembo and Jambo)	female	2	Fin&Csdx	Anti-androgen	S	21

#92 Jambo (lives with Tembo and Haji)	female	2	Fin&Csdx	Anti-androgen	D	40

The colony has been supported by the National Institute of Mental Health and, more recently, the National Science Foundation, for studies of the endocrine substrates of genital masculinization and dominance in the female spotted hyena [41,54]. Accordingly, the majority of subjects observed in the present study were treated with compounds that blocked the actions of androgens or estrogens *in utero*, or were gonadectomized at varying stages of development (see Table 1). Thus although the hormonal and developmental state of the animals were not a designed factor in our study, the effect of these treatments had to be examined so that they could be discarded (or alternatively further examined if they had showed interesting results). The animals in the study were initially assigned to the following groups: (A) "control" (no treatment or simple vasectomy; 2 males, 3 females), (B) treatment with "anti-androgens" (flutamide and finasteride, or Casodex and finasteride) *in utero *(3 males, 7 females; for details of drug treatments, see [55]); or (C) treatment with an aromatase inhibitor (Letrozole, preventing the synthesis of estrogen) *in utero *(1 male, 1 female; for details of Letrozole treatment see [56]). In addition, (D)1 male and 1 female were gonadectomized during the first months of life. When compared with control hyenas, gonadectomized hyenas would have had low concentrations of plasma testosterone, or estradiol, at the time of testing [57]. Male hyenas treated with anti-androgens in utero have reduced concentrations of testosterone as adults [58]; while female spotted hyenas treated with anti-androgens in utero displayed elevated concentrations of estradiol [58]. In Hyenas, males are mature at 2 years old, and females at 3 (Glickman, *pers. obs*.).

To test the effect of hormonal treatments, we performed statistical analyses by dividing the animals into the four groups mentioned above. Alternatively, we analyzed the data by dividing the experimental animals into two groups (anti-estrogen and anti-androgens) by including the two gonadectomized animals into these groups (the female into the anti-estrogen and the male in the anti-androgen group). Finally, we also tested the control animals against all experimental animals. These various groupings were done to potentially increase the statistical power of the tests given the small sample size in each group. As described below, none of the analyses revealed an effect of hormonal treatment.

### Vocalizations recordings

We recorded hyena's vocalizations in the morning, prior to feeding (recording chain: Shure Model 16A condenser microphone placed at 3-5 meters from the vocalizing animal, connected to a MARANTZ PMD670 solid state recorder; sampling frequency = 44100 Hz; frequency response of the recording chain: ± 2.5 dB over the 50 to 15,000 Hz frequency range). Giggling was elicited by teasing the focused animal with a bone or a piece of meat presented through the fence of the enclosure. In most cases (and in particular for subordinate animals), the recorded animal was isolated from its cage-mate to avoid fights and potential injuries.

To limit the potential impact of pseudo-replication, each individual was recorded over a minimum of 4 different days (10 females, 7 males; see Table 1 for details). Giggles are emitted in bouts which consist of a rapid succession of very short calls or notes (Figure 1A &2). In this study, we focused our analysis on the information that could be extracted from both the average acoustical features of single notes and from features describing the range of giggle notes produced by single animals. Although, there is certainly additional information in the structure of a particular sequence of notes within bouts these were not examined in this analysis; analyzing such sequence effects would require the estimation of transition probabilities for particular note types and this initial data set is too small for such characterization. The notes used for the analysis were selected pseudo-randomly from the recordings to avoid noisy or transitional sounds such as whines within the giggles. The raw data consisted of 254 giggle bouts and a total of 1807 notes (Average notes/bout = 7.1, Standard deviation = 4.1). The final data set consisted of 41 ± 19 (std) notes per individual (minimum = 20; maximum = 98), or a total of 695 notes. Balanced statistical analyses were performed by repeatedly choosing 20 random notes for each animal.

### Sound analysis

To characterize the giggle notes' complex acoustic structure, we performed the measurement of 13 variables, in both temporal and frequency domains (Figure 1B, C). The acoustical analysis was done using custom software written in MATLAB (Ver 7.6, The Mathworks, Cambridge MA). The time-varying fundamental frequency of notes was extracted using a custom algorithm that combined two separate measurements of fundamental frequency to obtain a best guess using a Bayesian approach. The two methods were: 1) a cepstral analysis where the inverse time corresponding to the highest peak in the Fourier transform of the log spectrogram was taken as the fundamental, and 2) a direct analysis of the short-time Fourier Transform (STFT) of the sound waveform where the smallest distance between all major successive peaks including a peak at zero was taken as the fundamental. The spectrogram and the STFT where obtained with Gaussian shaped windows with a time-frequency scale of 3.2 ms/50 Hz measured by the standard deviation parameter of the Gaussian window in time or frequency. The Bayesian approach consisted of assigning a likelihood to each of these two measurements and a prior. The likelihood was obtained by estimating the probability of the possible fundamental frequencies given the relative size of the peaks in the cepstrum or STFT. The prior depended on previous history, more precisely on the local derivative of the fundamental obtained from the prior three measurements (the prior was uniform for fundamental below 1 kHz for the initial measurements). The algorithm returned the best guess of the fundamental and its probability. Guesses with low probabilities were dismissed as sounds lacking clear periodicity. The performance of the algorithm was verified visually for all calls in the database and was deemed to perform very similarly to what an expert acoustician might extract from analyzing the spectrogram. Using this methodology, we extracted the time-varying fundamental frequency of each note in our data set with a (over-sampled) resolution of 1 ms. The following parameters were then calculated from these data: mean of the fundamental frequencies ('Mean F'), maximum of the fundamental frequencies ('Max F'), minimum of the fundamental frequencies ('Min F') and the coefficient of variation of the fundamental frequencies ('CV F'). We also extracted acoustical parameters from the frequency spectrum obtained for entire notes. The frequency spectrum was calculated with the Welch average periodogram method using a 23.2 ms Hanning window [59]. From the frequency spectrum, we measured the frequencies corresponding to the first, second and third quartiles of energy ('Q1', 'Q2', 'Q3', respectively), the mean frequency ('Mean S'), and the standard deviation of the spectrum ('SD S'). We also extruded three additional measures of spectral shape: skewness, kurtosis and entropy. The spectrum skewness ('Skew') was calculated as ; the spectrum kurtosis ('Kurt') was calculated as , where *f*_*i *_= frequencies, p(fi) = the normalized power spectrum () and σ^2 ^is the variance of the spectrum given by . The spectral entropy ('Ent') was calculated as . These values of spectral shape have been recently proposed for acoustical analysis (Seewave R software http://rug.mnhn.fr/seewave[60]). Sound duration ('Dur') of each note was measured visually from the oscillogram and its corresponding spectrogram.

All measures described above are used to describe the acoustical structure of single notes, or when averaged to describe giggles notes produced by an individual, as a prototypical single giggle note. However, it is clear from experiencing giggle sounds (personal experience), or, for the reader of this article by visual inspection of spectrograms (Figure 1 &2) as well as listening to the example sound clips (see Additional Files) that individual hyenas modulate both the fundamental frequency (pitch) and the type of giggle note (timbre) that they produce. The structure present in the succession of giggles notes in a bout is rich, almost musical, and potentially very informative. In this study, we just began the analysis of this structure by calculating the coefficient of variation (CV) of the mean fundamental frequency ('CVMeanF') and of the spectral mean ('CVMeanS') from all the giggle notes obtained from each individual. In other words, for each hyena, we calculated from all its giggle notes, one CVMeanF and one CVMeanS. These measures provide a simple quantitative measurement of how much each individual varies the pitch and the timbre of the giggle note that it produces. Since the CV is obtained by dividing the standard deviation by the mean, we also obtained the mean of 'Mean F' ('GrandMeanF'), the standard deviation of 'Mean F' ('SDMeanF'), the mean of 'Mean S' ('GrandMeanS') and standard deviation of 'Mean S' ('SDMeanS'). We examined these means and standard deviations to assess their respective contribution to the coefficient of variance.

### Statistical analysis

For all multivariate statistical analyses, the raw values of the 13 acoustical parameters of interest were centred and normalized (i.e. transformed into z-scores) to insure correct weighting since our acoustical parameters had different units (e.g., 'Mean F' and 'CV F'). Normality was visually inspected using the "normplot" command in Matlab.

**Figure 3:**
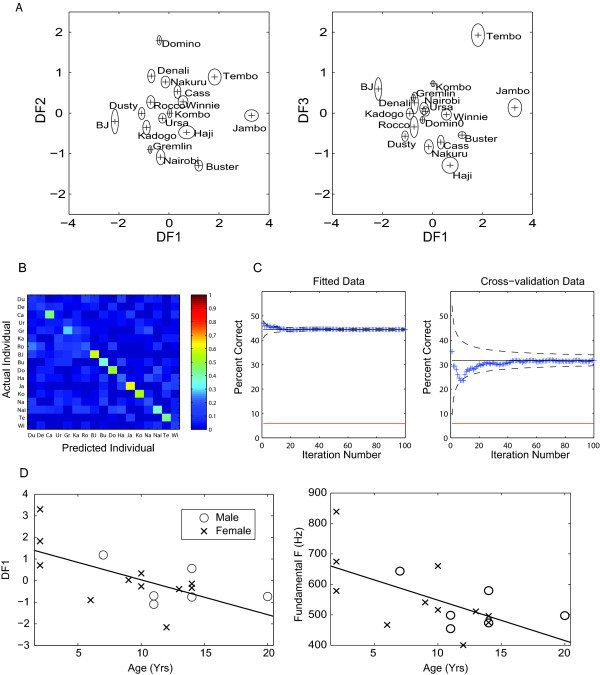
**Individual and age information in the gigglenote**. **A**. Position of individuals' centroids in function of the first three discriminant variables that maximizes individual separation. The left panel shows the centroids on a 2D projection for discriminant variables 1 and 2 and the right panel shows the centroids on a 2D projection for discriminant variables 1 and 3. The discriminant functions have been scaled so that the within-variance is 1; in other words, assuming normality, for each animal, 67% of its notes would be found in a sphere of radius 1 centred on these centroids. The plotted ovals around the centroids show one standard error of the mean. **B**. The confusion matrix obtained from the DFA on the cross-validation data set. The confusion matrix shows by colouring cell (i, j) the conditional probability of guessing that the test giggle notes came from individual j when in fact it was emitted by i. **C**. Average (cumulative) percent correct of calls classification according to the emitter's identity as a function of the number of random iterations for the data used to fit the discriminant functions (left) and for the data used for cross-validation (right). The dotted lines show two standard errors deviations from the final mean. The red line shows chance. **D**. Correlation between age and giggles' acoustic structure as described by the first discriminant function (left panel) and as described by fundamental frequency (right panel).

We analyzed the differences between giggles notes from different individuals or groups of individuals using Discriminant Function Analysis (DFA) [61]. The DFA was performed in Matlab (using the functions 'manova', and 'classify') and repeated in R for further validation as explained below. A DFA is composed of two steps: in the first step a set of discriminant functions is obtained from a training data set; in the second step these functions are used to test classification on a validation set. We chose the linear discriminant functions given by the eigenvectors of the ratio of the between and within covariance matrices; this approach is equivalent to a multivariate analysis of variance (MANOVA). The MANOVA assumes a jointly normal distribution for the parameters that describe the variability between and within individuals and returns the number of significant discriminant functions. The statistical significance of the functions is assessed by assuming normal distributions and calculating Wilk's lambda test statistic. In the second step, these discriminant functions are then used to classify giggle notes chosen from a validation data set. This cross-validation step gives a measure of the effect size (the percent correct) and of the statistical significance by comparing the percent correct and its standard error to chance. The measure of standard error is obtained by analyzing the percent correct assignment of 100 random selections of the original data set divided into a fitting and testing set. In all cases the training set consisted of 19 randomly selected giggle notes per animal and the discrimination was tested on a different randomly selected note (Note 19+1 = 20, the smallest number of notes that we obtained for an animal in our data set). As shown in Figure 3 and Results, 100 random selections were more than sufficient to obtain good estimates. By performing this cross-validation step, not only does one obtain a desirable measure of effect size (the percent correct) but also the assumption of normality is relaxed.

From the cross-validation results, we could also extract a complete confusion matrix: the conditional probability of guessing that the test giggle note came from individual i when in fact it was emitted by j:

From the confusion matrix, one can also obtain a measure of the goodness of the classification (effect size) by estimating the mutual information between guesses and actual values [62]:

Here the probability of the actual values, p(j), is uniform because the same number of testing giggles is used for each individual or group. When the DFA is used to classify individuals (n = 17), p(j) = 1/17 = 0.058. p(i, j) and p(i) are obtained from the confusion matrix and Bayes' theorem. It should be noted that the MI will have a positive bias for small number of testing notes and will have a negative bias (or be bounded) when the number of individuals is too small. For example, for 17 individuals, the upper bound for estimate of MI from the confusion matrix is log_2_(17) = 4.75 bits. As shown in the results, we were far from the regime were the negative bias becomes important and we corrected for positive bias effects. The measure of effect size provided by the MI is then independent of the number of animals or calls and, moreover, can be normalized by the length of the sound (by division) to get an effect size in bits/s. It can therefore be used to compare results on the information bearing content of communication sounds across experiments and species.

The straightforward DFA described above was used to evaluate the information present in giggle notes about individual identity. However, this procedure needs to be further modified when DFA is used to assess differences among groups of individuals classified by their age, social status or treatment effect. For those analyses, there are two additional statistical considerations: pseudo-replication and nested and/or interacting effects. The pseudo-replication comes from the fact that, when analyzing effects other than individual identity, giggle notes from the same animal are not statistically independent measures. In fact, the individual identity is a nested effect: one needs to consider differences in notes from different individuals as a separate effect that might explain some or all of the differences observed between groups. This point was clearly made by Mundry and Sommer [63] who suggested addressing the nested individual factor in a DFA by comparing the percent correct obtained in the analysis to the distribution of percent correct values obtained by randomly assigning the group identity to each individual. In our analysis, this distribution was obtained from 1000 randomly created data sets where the group identity of each individual is permuted in each set while preserving the number in each group. Note that in the random sets any information that is dependent on identity (such as belonging to a particular dyad) is also lost. This procedure is called permuted DFA (pDFA). The pDFA was performed in Matlab and also calculated with a R routine provided by Roger Mundry [63]. The statistical conclusions from both the Matlab and R routines were identical although we did observe small differences in the percent correct measures. In this study, we report the results obtained from the Matlab routines.

To further examine the potential interaction between the individual differences and the different conditions (age, sex, dominance, treatment), we performed a multiple linear regression (least-square and robust) with age, sex, dominance and treatment as predictors for the average value of the discriminant functions obtained in the DFA for individual differences. By using the average value for each individual in the regression we eliminated the pseudo replication problem that could lead to inflated significance. To minimize the number of statistical tests, this regression was performed for the first discriminant function, then the second, and so forth until we failed to find an effect. When age was a significant factor, we also used an analysis of covariance (ANCOVA) as an exploratory tool to investigate potential interactions between age and the other independent variables; in the ANCOVA age is treated as a regular scale variable in linear regression and the other conditions are examined to determine whether separate lines for each condition are warranted (the interaction effect). The p-values were then adjusted for multiple tests using the Bonferronni correction.

We also performed six linear regression analyses (least-square and robust) with 'GrandMeanF', 'SDMeanF', 'GrandMeanS', 'SDMeanS' "CVMeanF" and "CVMeanS" as dependent variables and age, sex, dominance and treatment as predictor variables. The p-values were corrected for multiple tests using the Benjamini and Hochberg False Discovery Rate [64]. ANCOVA was then used to examine the presence of interaction effects between age and other predictor variables when age was a significant factor in the linear stepwise regression analysis. Finally, considering that the dominance/subordinate status was assessed for each individual in respect to its cage-mate, we performed paired t-tests considering hyena dyads. For the single triad in our data (see table 1), Jambo was assessed as being dominant over his two cage mates, Tembo and Haji; thus, we took the average acoustical parameters of Tembo and Haji to characterize the subordinate call.

## Results

### Giggle note acoustic structure

All analyzed giggle notes (n = 695) showed the same general structure, i.e., short, broad-band signals, with some modulation in frequency (Figure 1B). Average note duration was 69 ± 18 ms (mean ± standard deviation). The mean fundamental frequency was 547 ± 146 Hz, with a maximum of 741 ± 180 Hz and a minimum of 399 ± 143 Hz. The fundamental frequency was modulated in time with a coefficient of variation of 0.155 ± 0.101. The energy was concentrated on the lower- harmonics as the frequency values at the first (25%), second (50%) and third (75%) quartiles of energy are 635 ± 398 Hz, 848 ± 556 Hz, and 1196 ± 717 Hz respectively. However, this distribution showed great variation among individuals. For example, the frequency at the third quartile ranged from 754 ± 315 Hz to 2279 ± 996 Hz, depending on individuals. The spectrum mean frequency was 979 ± 495 Hz, with a mean standard deviation of 544 ± 229 Hz. The frequency spectrum showed a positive skewness (skew equals 2.56 ± 1.93), underlying that most of the energy was concentrated over the low part of the signal frequency bandwidth. The leptokurticity of the spectrum (kurtosis of 18.5 ± 24.5) and the low values of spectral entropy (0.52 ± 0.12) quantify the fact that the energy was concentrated at a few frequency values (peaks). Differences in giggle notes from different individuals are illustrated in Figure 1A and 2 where giggle bouts from four individuals are shown: as can be seen the two bouts coming from the same individual bared similarities while two bouts coming from different individuals were clearly more distinct.

### Acoustic Structure in Succession of Giggle Notes

Giggle notes are produced in bouts as shown in figures 1 and 2. In our experiment, a typical giggle bout lasted less than 1 second and had approximately 7 notes (Avg = 7.1 notes/bout). The longest bout we recorded lasted 4s and had 28 notes. The shortest had two notes and lasted 200 ms.

The sequence of notes in a giggle bout has also a rich acoustic structure, in the sense that both the pitch and the timbre of giggle notes appear to change in an orderly fashion from the first to the last note in a bout. In this study, we coarsely began to quantify this structure by examining the variability in the mean fundamental and the spectrum mean frequency for each individual. The values for those two measurements are shown in the top panels in Figure 1A for the succession of giggle notes for the first example bout for each animal. In these examples, Ursa varied the mean fundamental (F) and the spectrum mean frequency (S) across its giggle notes more than did Kombo. It can also be clearly observed in the spectrogram that within a giggle bout, Ursa used more different "types" of notes than Kombo (see also the corresponding example sound clips). The same difference can be observed between the giggle notes produce by Winnie (more variable) and Kadogo shown in figure 2. Ursa and Winnie were subordinate animals while Kombo and Kadogo were dominant animals. Ursa and Kombo (shown in figure 1) were control animals while Winnie and Kadogo (shown in figure 2) were treated with anti-androgen and anti-estrogens respectively. We will show below that dominance status and age both affect the variability of giggle notes within animals but that hormonal treatment did not appear to affect the acoustic structure of giggle vocalizations as described by the measures we used. Across all animals, the mean CV for the mean fundamental was 0.19+- 0.05 (Range: 0.12-0.34). The mean CV for the spectrum mean frequency was 0.35+-0.1 (Range: 0.22-0.57).

### Individual identity and age of the sender

The DFA identified significant acoustic differences between individuals, calculating eight significant linear discriminant functions that allowed maximizing individual separation (Figure 3A). Table 2 shows the variance explained by each of the eight functions and the variable loadings on each function for the three most important factors. The spectrum mean frequency and the mean fundamental were the two main factors that separated individuals on the first function. The second function relied on the spectrum standard deviation, the note duration and the third quartile of energy, while the third function depended on the spectrum standard deviation, the spectral entropy, and the mean fundamental. Thus, while the first function mostly reflected the fundamental frequency (pitch), the second and third function quantified the spectral envelope (one of the factors that affect timbre).

**Table 2:** MANOVA results.

Sorted Dimension	P	% Variance	Function Loadings
**1**	< 10^-3^	42.1	0.860 Mean S + 0.676 Mean F + 0.363 Min F

**2**	< 10^-3^	22.2 (64.3)	1.011 SD S - 0.787 Dur - 0.493 Q3

**3**	< 10^-3^	11.6 (75.9)	1.449 SD S - 1.200 Ent - 1.187 Mean F

**4**	< 10^-3^	8.2 (84.1)	1.393 Skew + 0.870 Ent - 0.831 Kurt

**5**	< 10^-3^	6.5 (90.6)	1.142 Skew + 1.001 CV F + 0.733 Q2

**6**	< 10^-3^	3.2 (93.8)	1.539 Kurt + -1.537 Max F - 1.520 Skew

**7**	< 10^-3^	2.6 (96.4)	-1.304 Mean F + 1.036 Q3 + 0.937 Q1

**8**	0.006	1.3 (97.7)	-7.824 Mean S + 3.597 Q3 + 2.053 Q2

The table shows the loading factors for the 8 significant linear discriminant functions that can be used to classify giggles notes by individual. Since the discriminant analysis is performed on z-scores and the functions are normalized so that the within-individual variance is 1, the coefficient of the loadings can be used to measure the effect of each acoustical parameter. The % variance is the percent of the between-individual variance of means that is explained. The number in parentheses is the cumulative variance explained.

The results of the cross-validation step showed that, although perfect individual identification was not achieved, the rate of success was greatly above chance (32% versus 6%) (Figure 3C). This is not surprising given the high number of significant acoustical parameters. However, there was also extensive variation in the classification success rate across individuals (average = 32%, individual range from 6% to 62%). In other words, in our sample, some animals appeared to produce similar giggle notes (e.g see Kadogo, Rocco and Nakuru in Figure 3A and 3B) and were thus not easily classified while other animals produced relatively unique vocalizations (e.g. Jambo, Kombo and BJ in Figure 3A and 3B).

To obtain another measure of effect size that could be used to compare with other studies, we also calculated the mutual information from the confusion matrix that shows the joint probability of the actual and predicted individuals in cross-validated data (Figure 3B). The mutual information was 1.09 bits (well below the ceiling value or log_2_(17) = 4.08 bits). Since this information was obtained for single giggle notes of average duration of 69 ms, the resulting baud rate is 15.8 bits/s. A mutual information of ~1 bit/giggle means that a giggle note carries the same amount of information as a variable that would be able to perfectly divide any hyena as belonging into one of two groups. For giggles, this same amount of information actually allows one to associate the hyena as belonging to one of many groups but not with 100% accuracy (for example in our study as 1 of 17 individuals but with 37% accuracy) [62].

The post-hoc linear regression with age, gender, dominance and treatment as predictor variables and the first discriminant function as a dependent variable was not statistically significant (F(4,12) = 2.4, p = 0.1) but we noted that the coefficient for age was significant both for the least-square and robust regression methods (p = 0.01 and p = 0.03 respectively). Older animals had lower values for the first function than younger animals (Figure 3D, left panel), which, given the loadings of this function, appears to reflect a lowering of the fundamental with age (see Figure 3D right panel and also below). The ANCOVA confirm the statistically significant effect of age (F(1,15) = 10.4, p = 0.005) but showed no statistically significant interaction between age and the other conditions (sex, dominance, treatment). The linear regression did not reveal any statistically significant factors for the second discriminant function and the other discriminant functions were therefore not tested to minimize the number of tests and prevent the risk of false positives. Thus, while the first function might be used to classify individuals based on their age, the other discriminant functions capture idiosyncratic features of each individual's giggles.

In accordance with these previous analyses, the multiple linear regression performed on 'GrandMeanF', 'SDMeanF', and 'GrandMeanS' showed an influence of age on these parameters (see Table 3), and thus confirmed the age-linked lowering of the giggle note mean fundamental frequency. From the age of 2 to 20 years-old, the mean fundamental frequency dropped from 650 Hz to less than 450 Hz (i.e. -30%). The linear regression line for that relationship is shown on the right panel of figure 3D (t(15) = -2.93, p = 0.01).

**Table 3:** Multiple linear regression results.

	Mean F	SD F	CV F	Mean S	SD S	CV S
**R^2^**	***0.56***	0.47	0.40	0.58	0.70	***0.71***
F(4,12)	***3.87***	2.69	2.00	4.19	7.16	***7.37***
p	***0.032***	0.082	0.157	0.024	0.0035	***0.0031***
p-FDR	***0.048***	0.098	0.157	0.048	0.0105	***0.0186***

**Age**			N. S.			
b(Hz/yr)	***-14.7 ***	-4.0		-50.4	-32.6	-0.01 (no units)
t	***-3.3***	-2.63		-3.7639	t = -4.97	-4.04
p	***0.006***	0.02		0.0027	0.0003	0.0016

**Dom**	N.S.	N. S.	N. S.	N. S.	N.S.	
t						***2.93***
p						***0.012***

**Sex**	N.S.	N. S.	N. S.	N. S.	N. S.	N. S.

**Treat**	N.S.	N. S.	N. S.	N. S.	N. S.	N. S.

Age*Dom			Not done			
F(1,13)	3.24	0.01		2.4	0.09	***5.07***
p	N.S.	N.S		N. S.	N. S.	***0.04***

The table shows the statistical results of the linear regression analysis performed for the mean fundamental (Mean F), the standard deviation of the fundamental (SD F) the coefficient of variation of the fundamental (CV F), the mean spectral density (Mean S), the standard deviation of the spectral density (SD S) and the CV of the spectral density (CV S) as separate dependent variables and age Dominance (Dom), Sex, Treatment (Treat) as predictor variables. The p-values are also shown corrected for multiple testing using the false discovery rate adjustment. Age is correlated with both the mean fundamental frequency and its standard deviation; they both decrease as the animal ages. The mean, SD, and CV of the mean spectral value (S) also decrease with age. The Age*Dom shows the results for the interaction between age and dominance performed with the post-hoc ANCOVA. The cells shown in bold italic are the significant results that are illustrated in the figures of the paper. The linear regression between Mean F and Age is shown in figure 2D and the regression and interaction between CV of S and Age and Dominance is shown in figure 3B. N.S. = Not significant.

### Hormonal treatment

The three hormonal treatments were found to result in distinctive giggle notes when the data were analyzed with the MANOVA (P < 10-3; DF(1) = 1.135 CV F + 0.688 Ent - 0.527 Max F F; DF(2) = 0.925 Max F + 0.823 SD S - 0.793 CV F) but the pDFA showed that these difference were primarily the result of individual differences. The pDFA showed that 48% of the notes in the cross-validation set could be classified correctly (versus chance at 25%) but similar performance or better could be achieved with four random groups 12% of the time. Similar non-conclusive results were found when experimental animals are classified along two treatment groups (anti-androgen vs anti-estrogen) or lumped into one group (see methods). With two treatment groups the pDFA yielded a 68% of mean correct assignment versus 55% chance but p = 0.59. The step-wise linear regression analysis for the CVMeanF, CVMeanS, GrandMeanF, SDMeanF, GrandMeanS and SDMeanS showed no effect of treatment (Table 3). Therefore, although we cannot exclude the possibility that treatment had an effect on the acoustical quality of the giggles, if this effect exists it is small and we do not have enough power in our experiment to measure it or it is present in acoustical features not characterized here.

### Dominance and Sex

**Figure 4:**
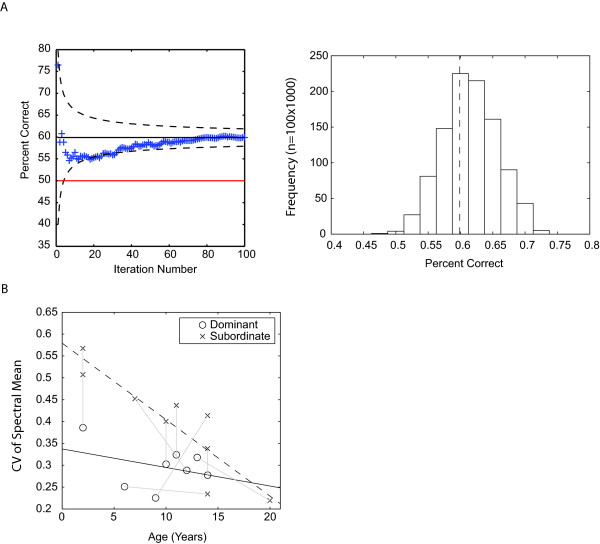
**Dominance information in the giggle note**. **A**. Left panel. Percent correct of calls classification according to the emitter's dominant/subordinate status as a function of iteration for cross-validation data. The cumulative average percent correct is plotted as a function of the iteration number. The solid red line shows chance and the dotted black lines the standard error of the estimate. Right panel. Distribution in the percent correct that is found by randomly assigning individuals to different groups. Although the discriminant function is significant and yields classification rates above chance, the permutations show that this successful classification could be solely due to individual differences and not to dominant/subordinate status. **B**. Coefficient of variation (CV) of the spectrum mean frequency calculated from all giggle notes for each individual. The CV is higher in subordinate animals than in dominant animals and decreases with age. The grey lines link the animals that were housed together and form the dyadic dominant/subordinate pairs.

The DFA found one significant linear discrimination function that could be used to classify giggle calls into dominant/subordinate groups (P < 10^-3^; DF(1) = 2.259 Mean S - 1.392 Q2 - 0.956 Q3). All the variables loading on this function dealt with the distribution with energy among the spectrum or, in other words, the spectral envelope. However, the pDFA showed that the successful classification was due to the nested effect of individual differences. Although, the cross-validation data showed that notes could be classified correctly 58% of the time (versus 50% for chance), the random permutations show that the same classification rates would be obtained 82% of the time (i.e. P = 0.82) in random assignments of individuals to two arbitrary groups (see figure 4A). Thus the results from pDFA did not support the idea that dominance status can be extracted from the acoustical characteristics of single giggles but instead showed that there is enough individual information in giggle notes that particular acoustical dimensions can be found to coarsely separate our 17 hyenas into two arbitrary groups.

On the other hand, the analysis of acoustical structure across giggle notes of single individuals showed a robust effect for dominance: the linear regression analysis showed that the coefficient of variation of the mean spectral frequency ('CVMeanS') calculated from all giggle notes for each individual was higher (ie. giggle notes are more variable) in subordinate animals than in dominant animals and that this effect decreased with age (see Figure 4B and Table 3). The ANCOVA post-hoc analysis confirmed that this interaction effect was significant (see Table 3). When the data was analysed as 8 dyads (and without taking age as a factor), a paired t-test also showed a significant effect for 'CVMeanS' (mean CVMeanS difference = 0.082, t(7) = 2.387, p = 0.048) but not for any other acoustical measure.

Sex was found to be distinctive by MANOVA (P < 10^-3^; DF(1) = 1.262 Ent -1.228 Q2 + 1.119 Mean S) but the pDFA shows that this separation could also be due to individual differences. In the cross-validated data sets, we found that 57% of notes could be classified according to gender but permutations show that this level of performance would be found 87% of the time. The stepwise regression analysis for the CVMeanF, "CVMeanS" 'GrandMeanF', 'SDMeanF', 'GrandMeanS' and 'SDMeanS' showed no effect of sex (Table 3).

## Discussion

### Giggles bear information about the sender

According to the analysis presented above, the hyena's laugh potentially encodes information about individual identity, dominant/subordinate status and age, giving receivers cues to assess the social position of an emitting individual. The discriminant functions used to separate individuals support the idea that information about individual identity is primarily encoded by pitch (1^st ^discriminant function) and by the energy distribution among the frequency spectrum of giggle notes (2^nd ^and 3^rd ^discriminant functions); note duration was also involved (2^nd ^discriminant function). As the first discriminant function correlated with age, pitch was age-dependent. Following our results, the pitch aspect of an individual signature is likely to change when the animal gets older. Besides this, age independent identity and other individual characteristics are mainly encoded by the energy distribution among the frequency spectrum. The dominant/subordinate status appears to be encoded by variations of the spectrum mean frequency within giggles of an individual. The fact that giggle notes are emitted in bouts thus represents a crucial aspect for the potential assessment of this social cue. That way, the multiple giggle notes could not only provide redundant information but also additional (synergistic) information from the structure created by particular note sequence.

We did not find reliable effect of sex even after correcting for the age effect. This result is not universal in hyena vocalizations: an analysis of groans by our group (*in prep*.) showed a very clear difference between males and females with males showing *higher *pitch than females. Giggles might therefore be less sexually dimorphic than groans and potentially more influenced by other factors. In addition, it is possible that pre-natal hormonal treatments also affected sexually dependent characteristics.

From a methodological point of view, the results from the classical and pDFAs show that while, information coding of age and individual identity within the giggle note are quite robust, any information that we uncovered about gender, dominant/subordinate status or hormonal treatment could be due solely to individual effects. Our data set and analyses therefore illustrate the importance of a careful use of DFA statistics of acoustic features when searching for vocal signatures, a point that was first stressed by [63].

### Individual identity coding

Previous studies, investigating gregarious mammals' and birds' acoustic signals in the context of mate or parent-offspring recognition, have shown that individual identity can be coded by one or several of the following sound features: the fundamental frequency ("pitch"), the energy distribution among the spectrum ("vocal timbre"), the characteristics of the frequency modulation of the fundamental (e.g., penguins: [65]; gulls: [66]; shearwaters: [67]; marmots: [68]; fur seals: [69,70]). All the acoustic signals supporting individual identification are usually highly redundant with regards to the coding process and thus resistant to masking effects. For example, non-nested birds use a secured code for long-range propagation of sender's identity that relies on lower harmonics band and frequency modulation of the fundamental. Both cues resist well to propagation-induced modifications such as filtering or scattering by obstacles. In nest-building species where acoustic exchanges occur mostly at short range, frequency modulation is almost absent and individual identity is encoded by pitch and energy distribution among the spectrum, the latter being highly sensitive to frequency-filtering during long-range propagation [65]. The present analysis showed that giggles encode individuality mostly by these two last cues, the frequency modulation being not primarily involved. This contrasts with another hyena vocalization, the whoop, where individual signature should be encoded by the pronounced frequency modulation, as visual inspections of spectrograms showed [51]. Thus and in spite of instabilities in their frequency spectrum [71], whoops might be more reliable labels of individual identity than giggles over long distances.

### The reasons for giggling: a puzzling situation

Up to now, no systematic study has tried to decipher the behavioural context in which giggles are emitted, nor their role in the society network. Field researchers have observed that these vocalizations are often produced during food contests, by animals that are prevented from securing access to the kill by the intervention of higher ranking individuals. More generally, giggling can occur in non-feeding situations, as the result of simple threat from another individual. Giggles have thus been considered as submissive signals [39,49]. The present study was done in captivity and our behavioural observations were certainly biased. Nevertheless, we observed that hyenas were giggling rather as a result of frustration (we kept the bone or the piece of meat out of their reach) than of harassment or chase.

Although they are emitted during close-range interactions between two or three individuals, giggles are loud and propagate over great distances [39](the authors *pers. obs*.). They are thus extremely susceptible to eavesdropping by remote receivers. Giggles may attract other spotted hyenas as well as lions *Panthera leo*, and even vultures [39]. During his study in the Ngorongoro (Tanzania), Kruuk [39] noticed that lions often eat prey previously killed by hyenas. Giggles could benefit an individual hyena is different situations. It is known, that a solitary hyena has no chance when confronted to a lion, whereas a hyena group often can "mob" one or two lions, and maintain or gain access to a carcass [72]. Thus a lone hyena encountering a kill dominated by lions could use its giggle call to rally its clan. The attraction of a neighbouring hyena clan or a lion group can also be an issue, as intense competition and giggling may occur over a kill currently mob by the giggling hyena's clan. In this situation, one can hypothesize that a dominant hyena might allow a subordinate hyena access to food to prevent it from giggling and attracting further attention from unwanted competitors. Or potentially giggling by multiple subordinate animals (giggle chorus) could serve as a distraction for the more dominant animals. However, these hypothetical situations could also potentially incur cost for the emitters who risk being completely deprived of food if the competing clan or lion group takes over. Cooperation and competition are everyday components of a spotted hyena's life. When hearing a giggling individual, clan-mates hyenas could get some information about "who" is currently in a competitive situation (in terms of individual identity, age class, dominant/subordinate status) and decide to join the giggler, or conversely to ignore it or move away. On a larger scale, giggles from a hyena group could attract conspecifics, allowing more successful "mobbing" of lions. It is also interesting to note that the loud giggle call is absent from the vocal repertoire of the sympatric but less social Brown hyena, *Hyaena brunnea *[49]. Field observations and playback experiments are needed to determine the attraction potential of giggles towards allies versus intra- and inter-specific competitors, and to assess the cost-to-benefit balance of this vocalization. The possibility of monitoring the size and the composition of a feeding group from a distance should also be investigated to assess if a clan could get information about its neighbours by simply eavesdropping on their giggles. This information could also be of interest to researchers with conservation purposes.

### Giggles and the spotted hyena's repertoire: acoustic tools for a social network

Primarily emitted during common confrontations, such as those occurring over food, giggles may play an important role in spreading of individual-related information among clan-mates. The social organization of a hyena clan involves a matrilineal hierarchy. The majority of adult males are immigrants and they are "queuing" for social status, i.e., the most recently arrived male is at the bottom of the male hierarchy [46]. Since males depart from their natal clan and attempt to join a new clan when mature, the age information embedded in the giggle could be highly informative.

In our study, hyenas were housed in dyads (and one triad) where a dominant/subordinate ranking was quickly established. The giggle was also produced in a conflict with a human which we attribute to have a neutral social rank. Although the bi-modal grouping and our experimental protocol allowed us to clearly divide animals into subordinate and dominant, it is a situation which is far from the hierarchical rank that could be found in wild clans of 10 to 90 individuals. It is possible that animals in the "middle" of the rank produce different giggle bouts when these are directed to higher ranking versus lower ranking animals. This hypothesis would also imply acoustical structure that is dependent not on morphological characteristics but on the recognition of social context and the possibility of vocal plasticity. This hypothesis could be tested both in the field and in our captive colony by housing animals in larger groups.

The vocal repertoire of spotted hyenas is very large and most of the calls should play a role in the regulation of the social network. Status signalling is almost certainly not restricted to giggles. The whoop is used to transfer information to remote congeners and may allow individual identification of the sender as well as its current emotional state over distances up to 5 km [51,53,73,74]. Not only are whoop calls highly idiosyncratic making but it is also known that mothers will respond to the whoop from their own cub more frequently than to the whoop of a non-kin cub, demonstrating that the individual characteristics in the whoop sounds are recognized and utilized [73]. It is likely that close-range vocalizations like the grunt, groan and growl, are also multi-informative [[39], Page et al. *in prep*.]. The use of both 'public' and 'private' signalling [75,76], would enable hyenas to manage their social interactions with great precision. In this context, giggles might play a dual role, addressed to both nearby clan-mates and remote potential allies.

## Conclusions

Spotted hyenas demonstrate high cognitive skills, like their ability to recognize third-party relationships [77], a characteristic that has been found in a restricted number of animals (e.g., primates [78]; birds [79]). Their substantial vocal repertoire should play a very important role, by providing eavesdroppers with a number of important cues about the emitters' identity and characteristics. As the present study showed, the giggle is likely to carry a broad range of messages, certainly not all perfectly reliable, but sufficiently informative to play a role during social interactions. Information carried by vocalizations, together with chemical, tactile and visual channels [48,80], ensure to the spotted hyena an array of communication signals which underlie its complex social system. More research, with a particular emphasis on experimental studies with playback - or involving manipulations -, is needed to specifically determine whether the information bearing structure in giggle sounds described here is actually used by the spotted hyena and, more generally, to decipher the complex communication network of this species. Comparing the spotted hyena's communication system with the one of other, less social, Hyaenids [81] would also be of great interest and facilitate understanding of the relationships between sociality and animal signals.

## Authors' contributions

NM and FT conceived and coordinated the study. All authors performed the work. NM and FT drafted the manuscript. All authors read and approved the final manuscript.

## Supplementary Material

Additional file 1 **Example #1 of a giggle bout from Ursa**. Wave file of an example giggle bout from Ursa. The spectrogram of this call is shown on the top left panel of figure 1A. Click here for file

Additional file 2 **Example #2 of a giggle bout from Ursa**. Wave file of an example giggle bout from Ursa. The spectrogram of this call is shown on the bottom left panel of figure 1A. Click here for file

Additional file 3 **Example #1 of a giggle bout from Kombo**. Wave file of an example giggle bout from Ursa. The spectrogram of this call is shown on the top right panel of figure 1A. Click here for file

Additional file 4 **Example #2 of a giggle bout from Kombo**. Wave file of an example giggle bout from Ursa. The spectrogram of this call is shown on the bottom right panel of figure 1A. Click here for file

Additional file 5 **Example #1 of a giggle bout from Winnie**. Wave file of an example giggle bout from Winnie. The spectrogram of this call is shown on the top left panel of figure 2. Click here for file

Additional file 6 **Example #2 of a giggle bout from Winnie**. Wave file of an example giggle bout from Winnie. The spectrogram of this call is shown on the bottom left panel of figure 2 Click here for file

Additional file 7 **Example #1 of a giggle bout from Kadogo**. Wave file of an example giggle bout from Kadogo. The spectrogram of this call is shown on the top right panel of figure 2. Click here for file

Additional file 8 **Example #2 of a giggle bout from Kadogo**. Wave file of an example giggle bout from Kadogo. The spectrogram of this call is shown on the bottom right panel of figure 2. Click here for file
